# Supplementary material for: How Is the Nociceptive Withdrawal Reflex Influenced by Increasing Doses of Propofol in Pigs?
Source: Animals (Basel). 2024 Apr 2;14(7):1081. doi: 10.3390/ani14071081 (PMC11010981; doi:10.3390/ani14071081)
Supplement: Supplementary file 1 [file animals-14-01081-s001.zip › File S2.pdf]

P1 – D1

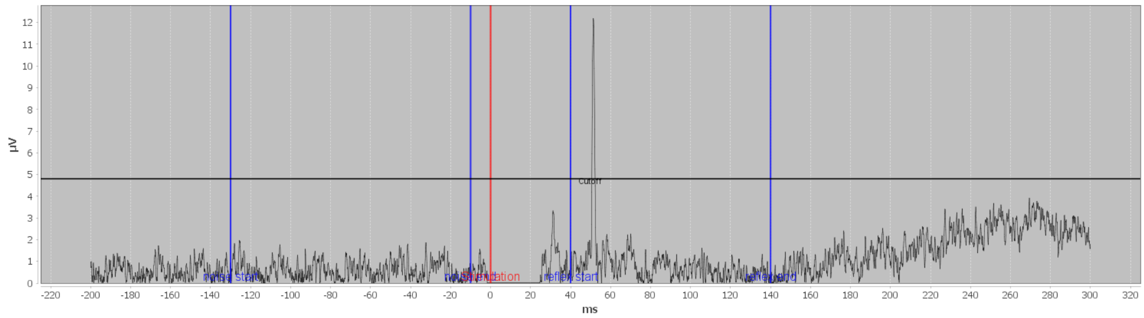

BSL

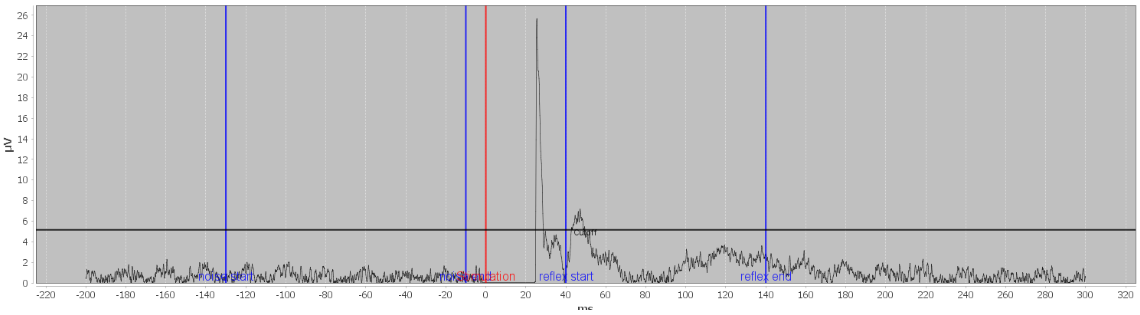

INT

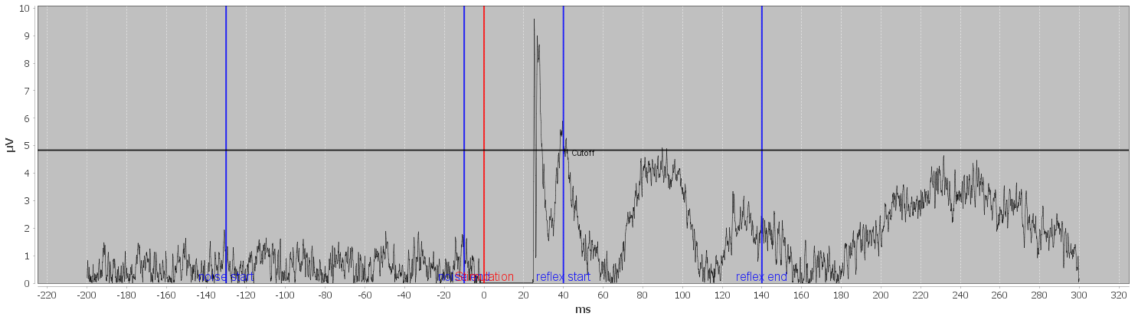

FI

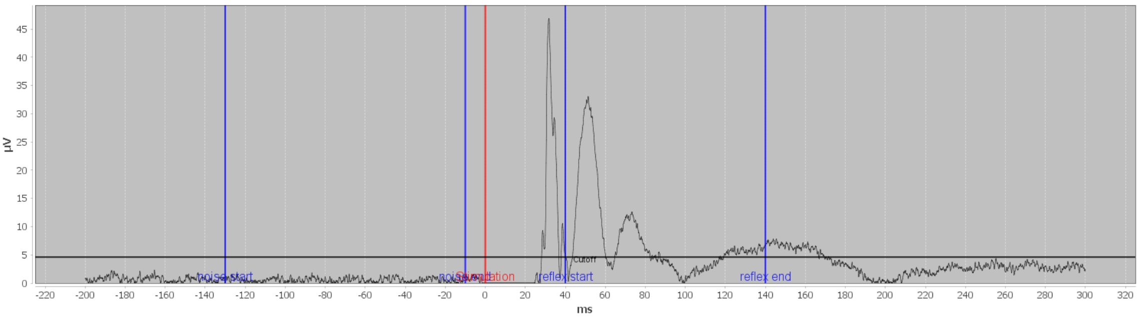

END

P1 – D2

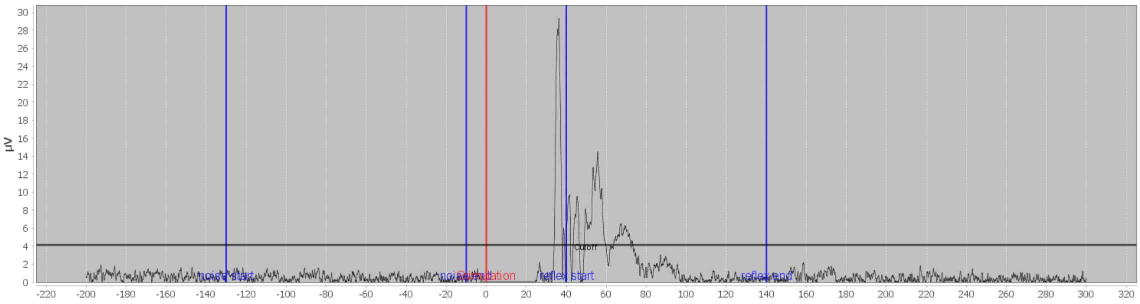

BSL

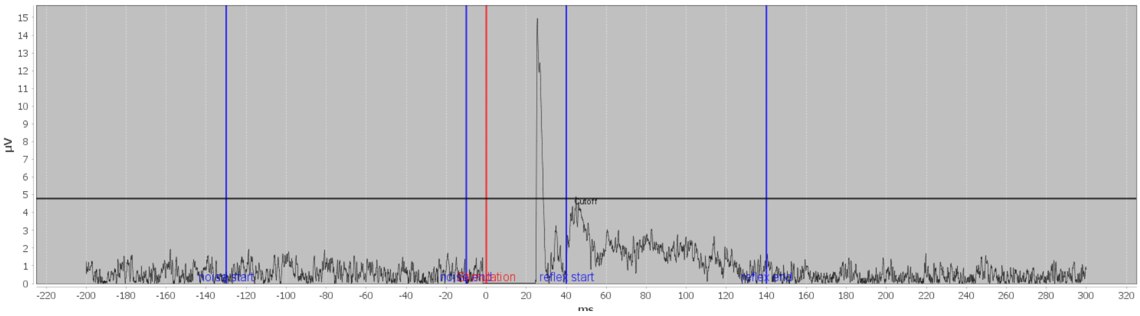

INT

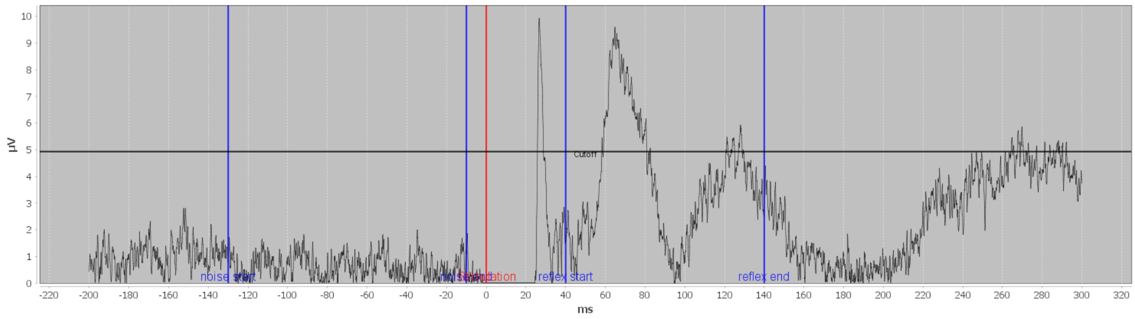

FI

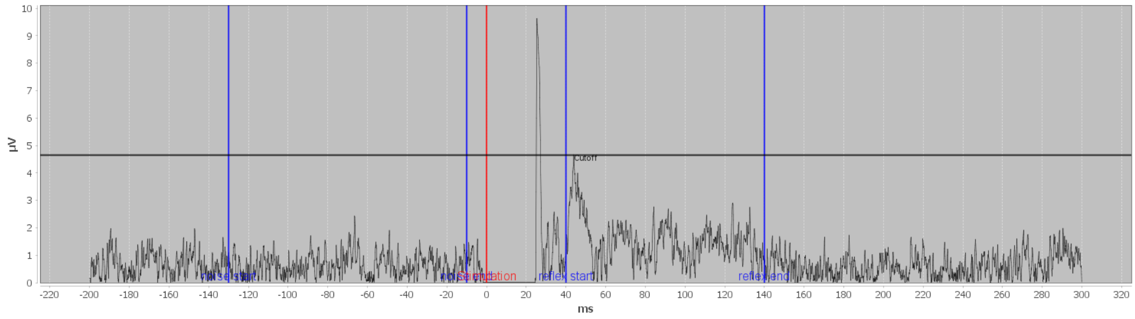

END

P1 – D3

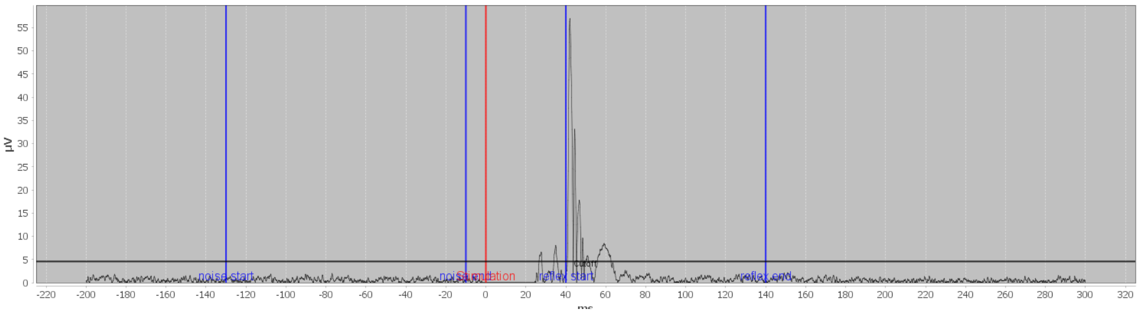

BSL

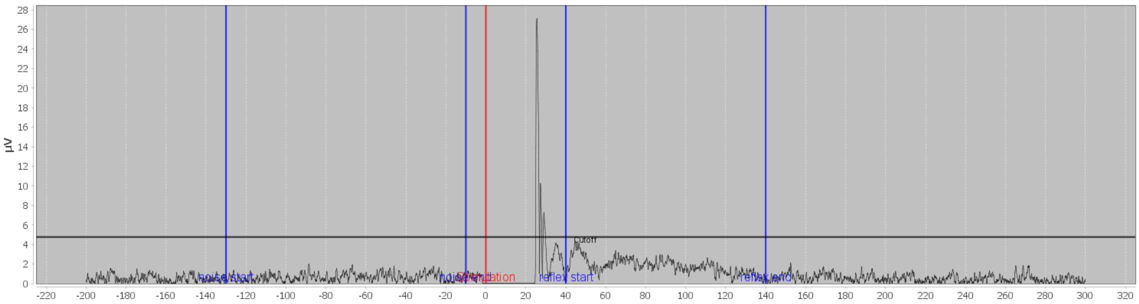

INT

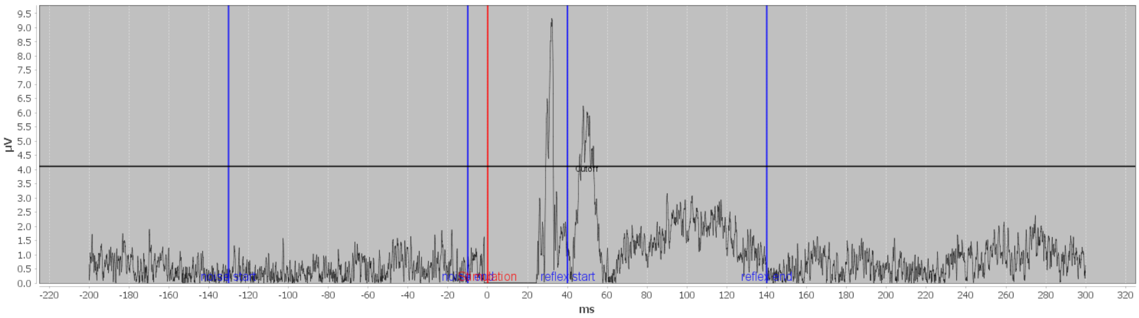

FI

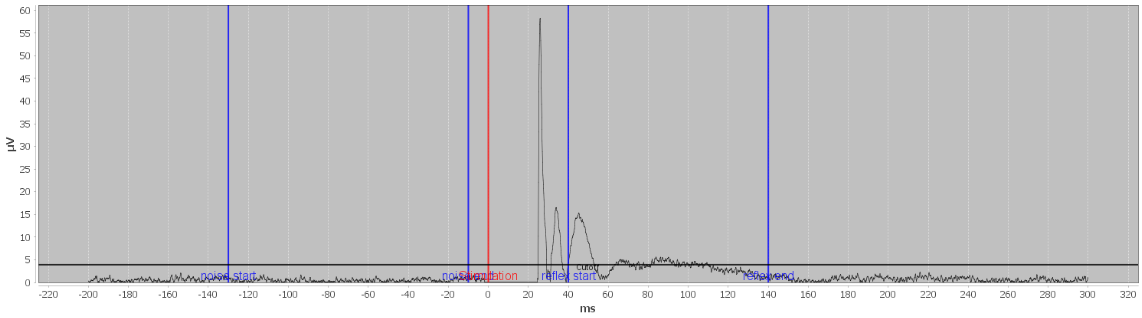

END

P2 – D1

Excluded (see text)

P2 – D2

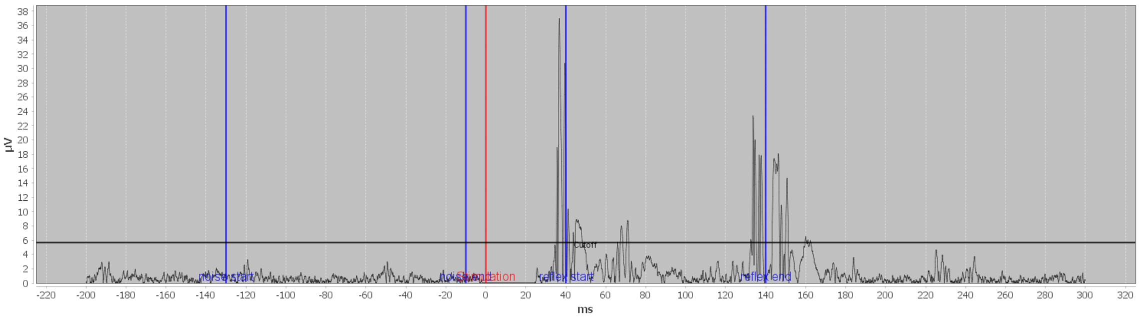

BSL

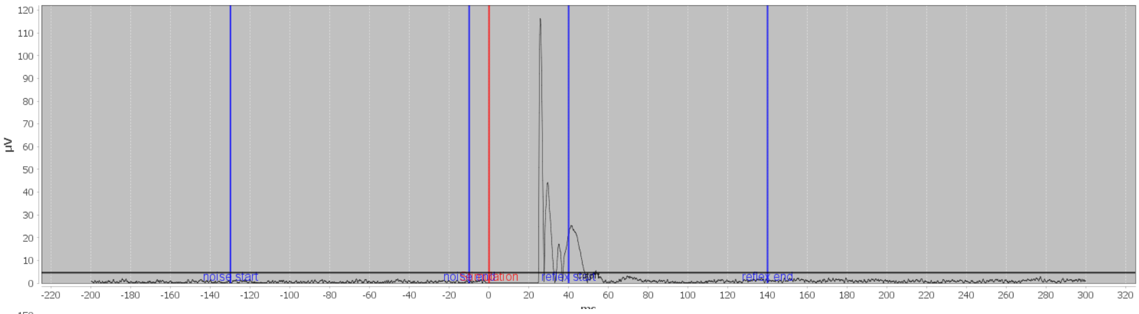

INT

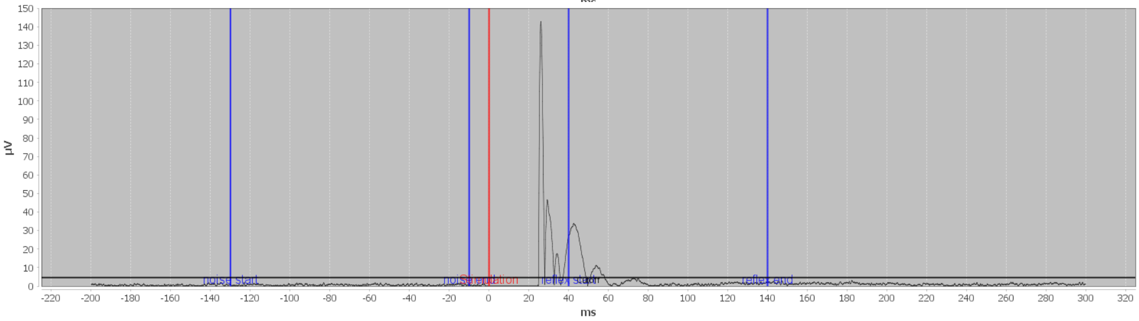

FI

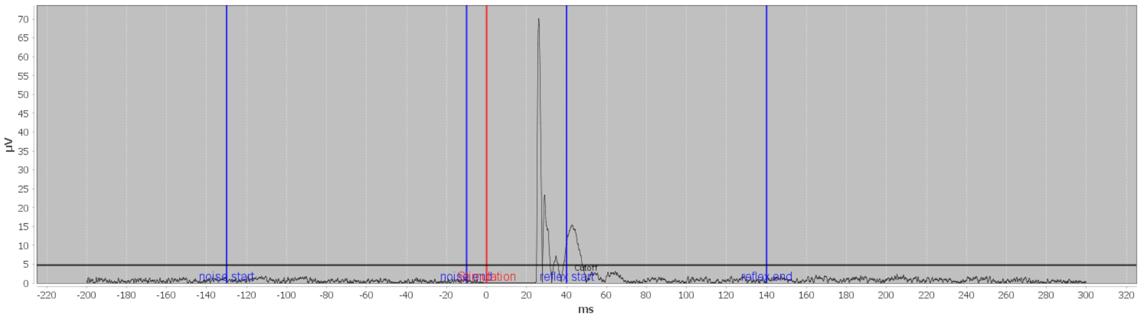

END

P2 – D3

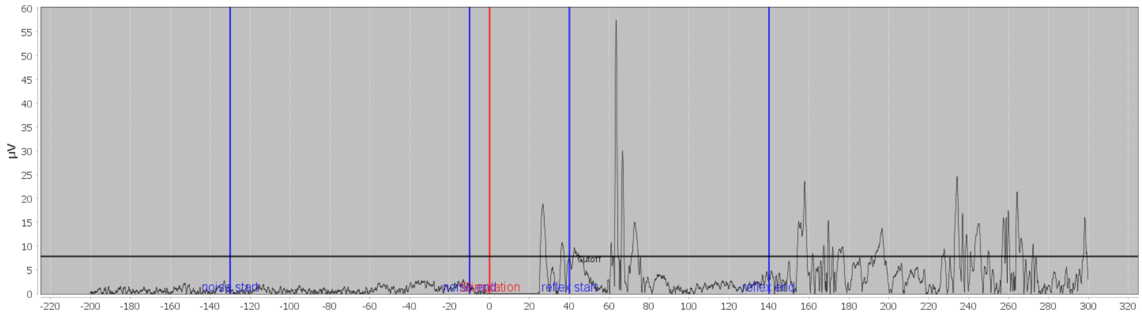

BSL

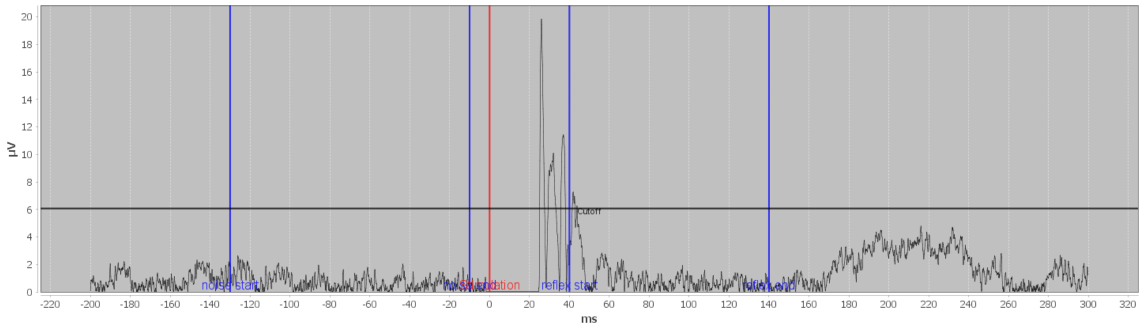

INT

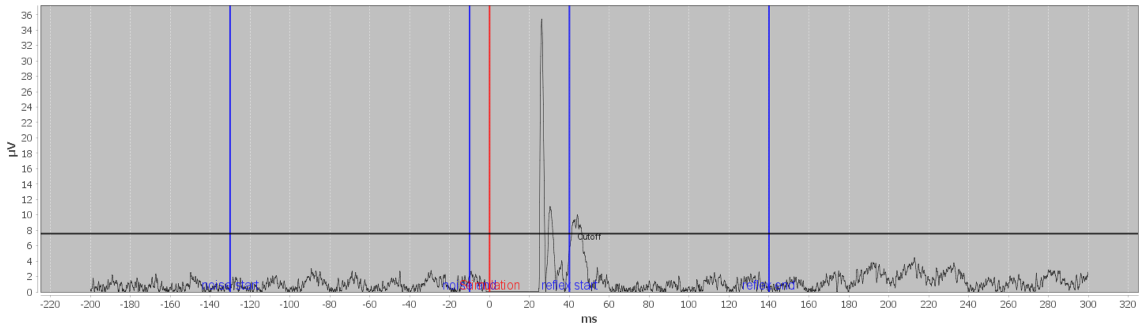

FI

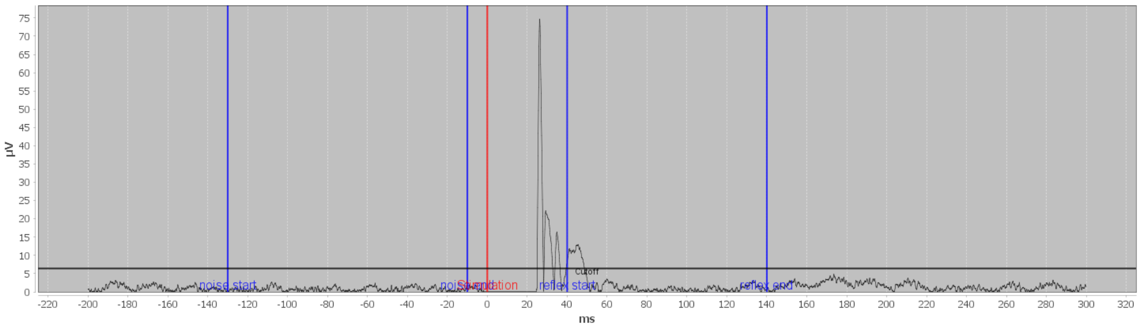

END

P3 – D1

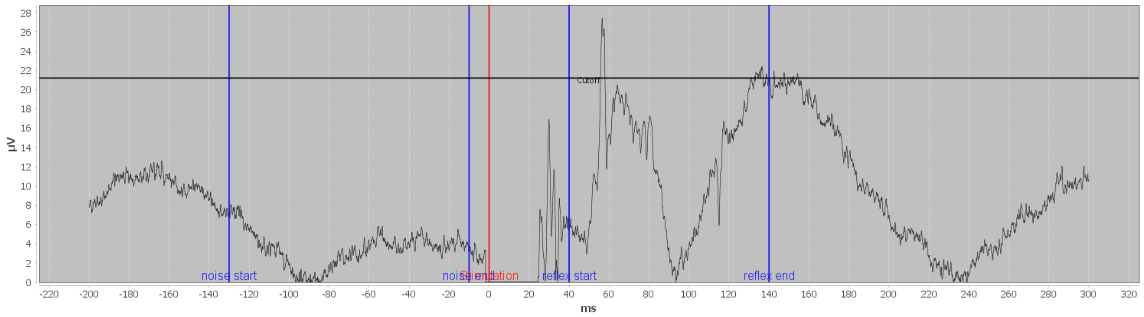

BSL

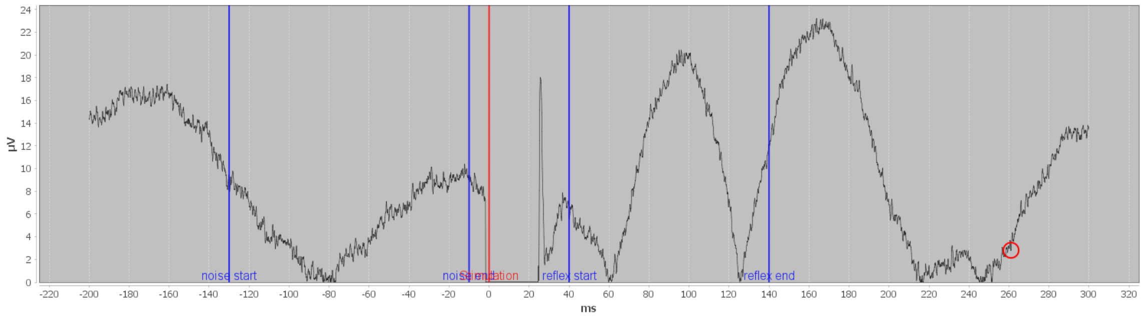

INT

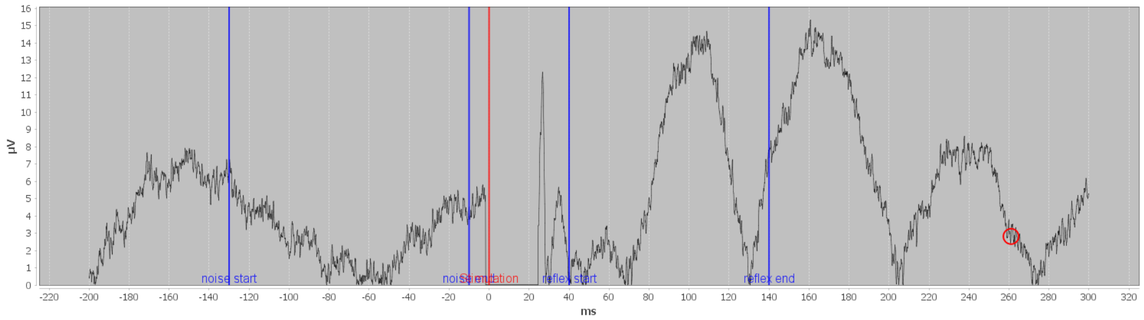

FI

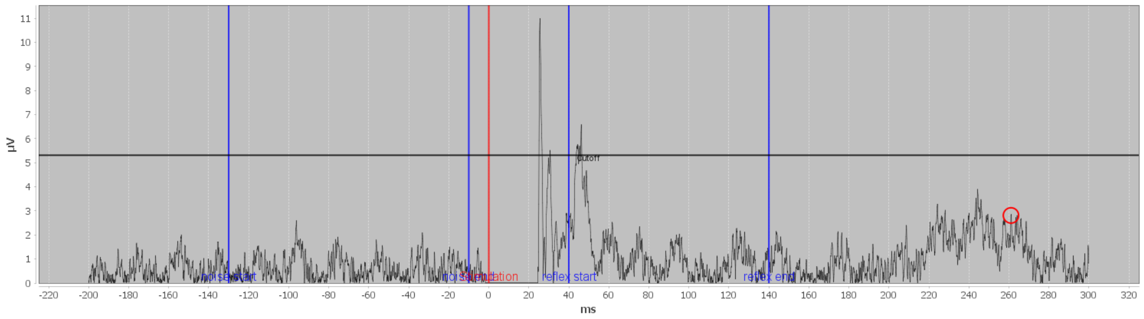

END

P3 – D2

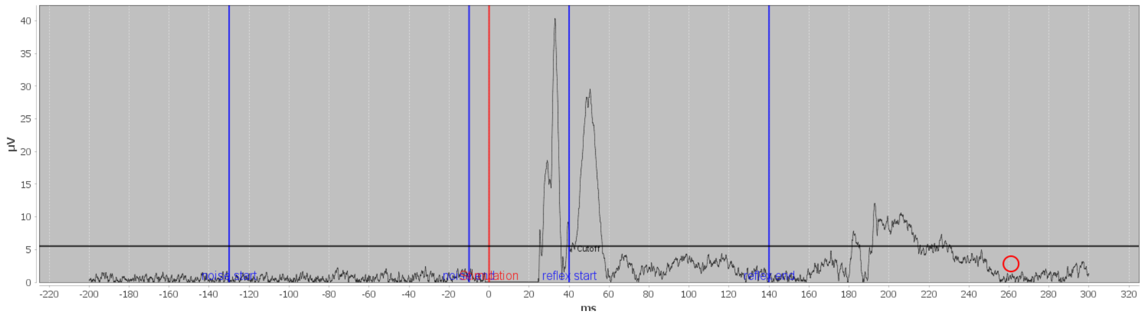

BSL

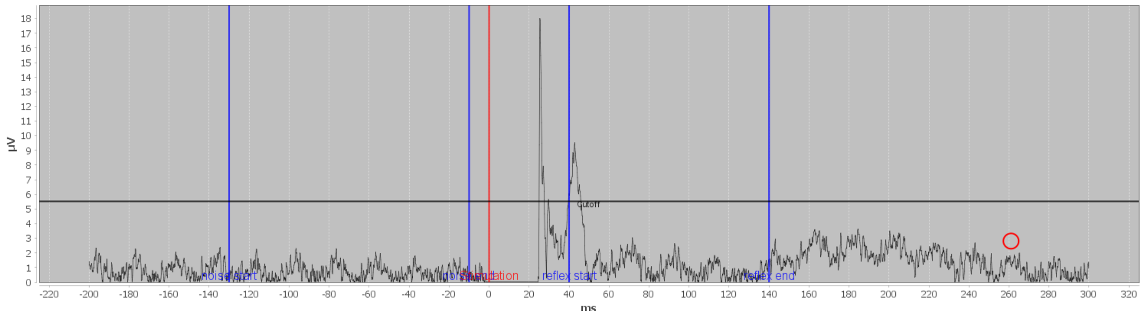

INT

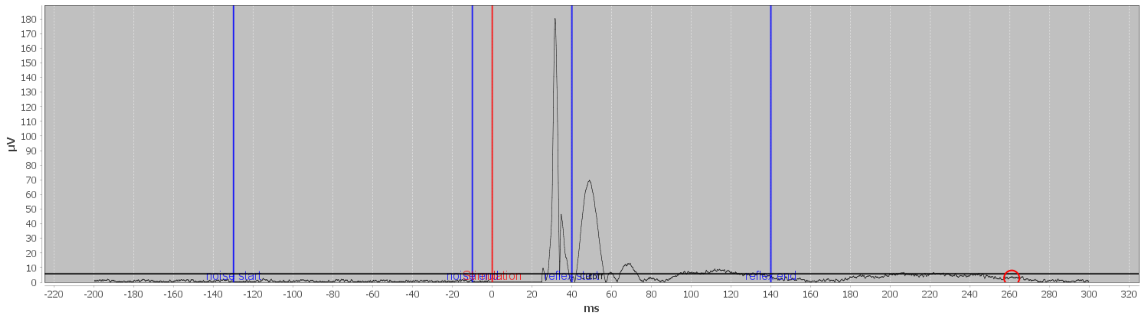

FI

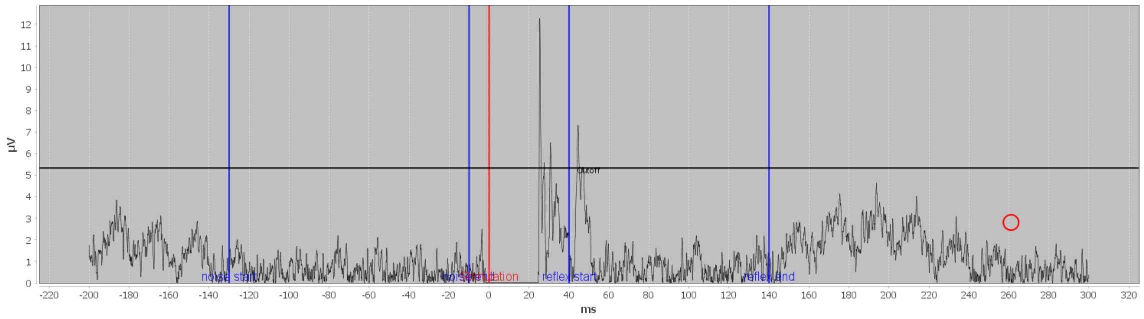

END

P3 – D3

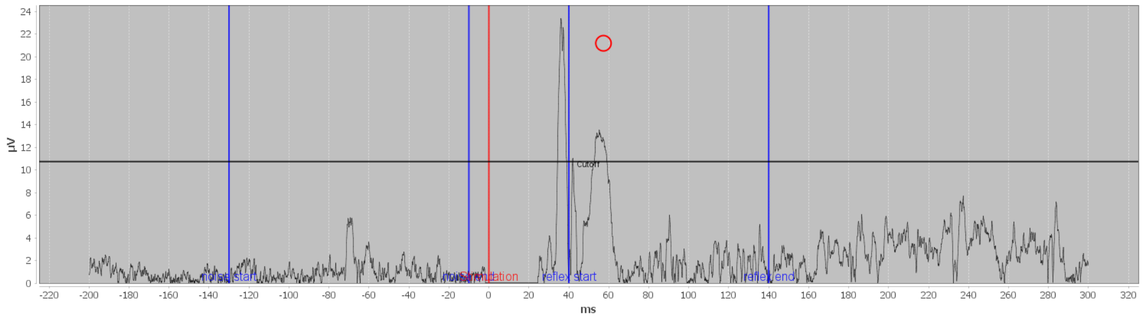

BSL

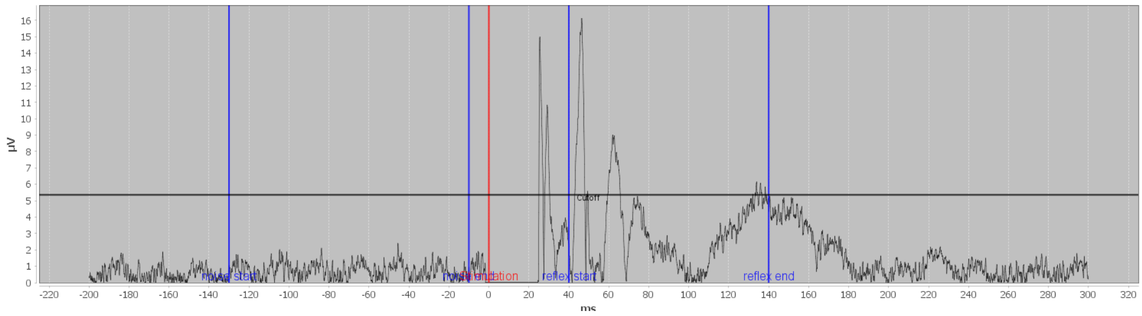

INT

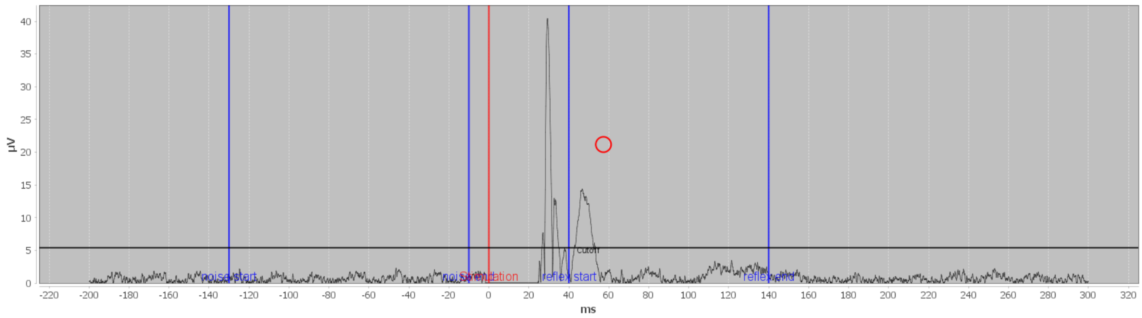

FI

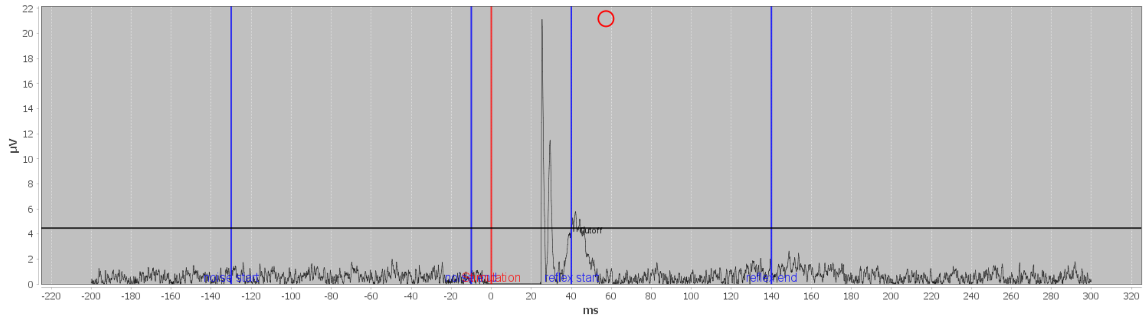

END

P4 – D1

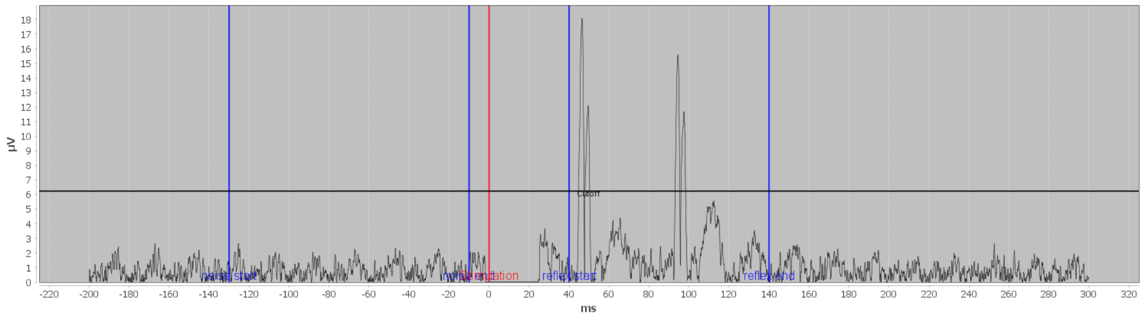

BSL

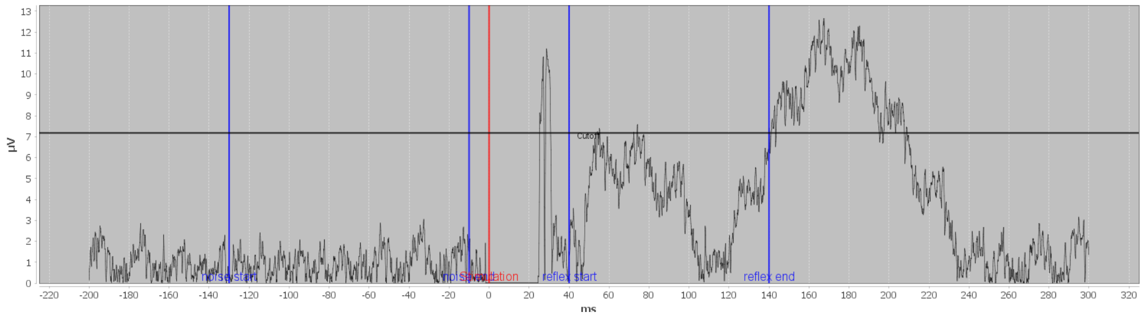

INT

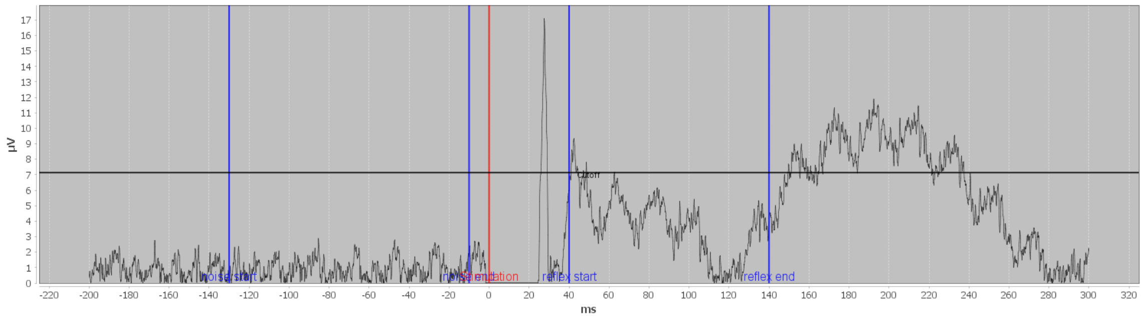

FI

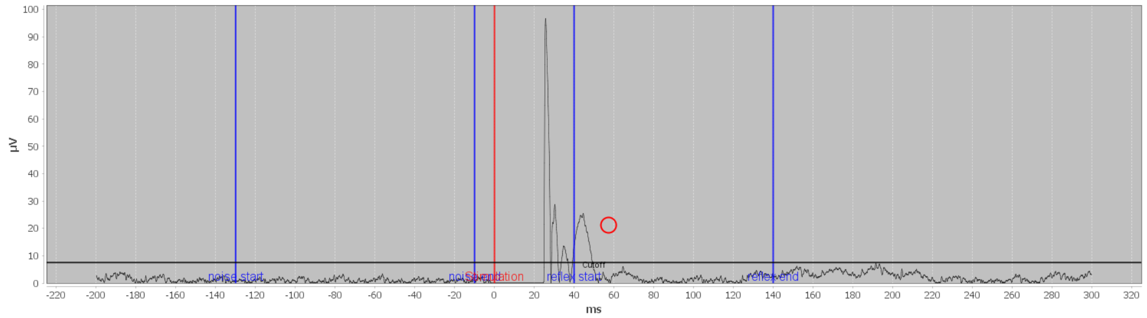

END

P4 – D2

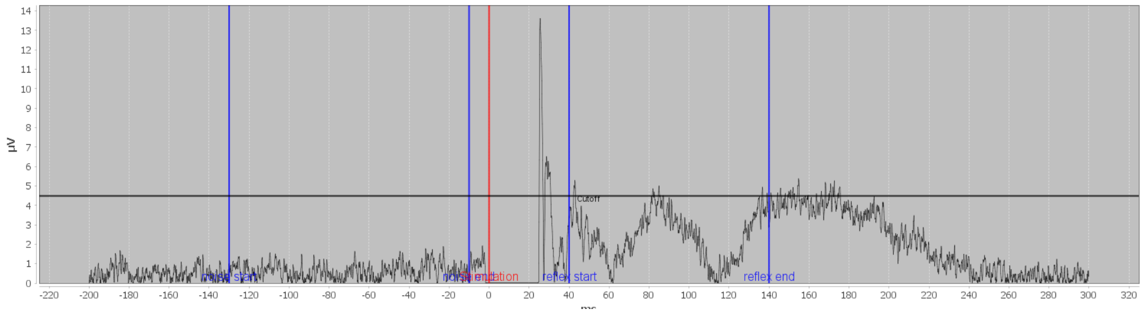

BSL

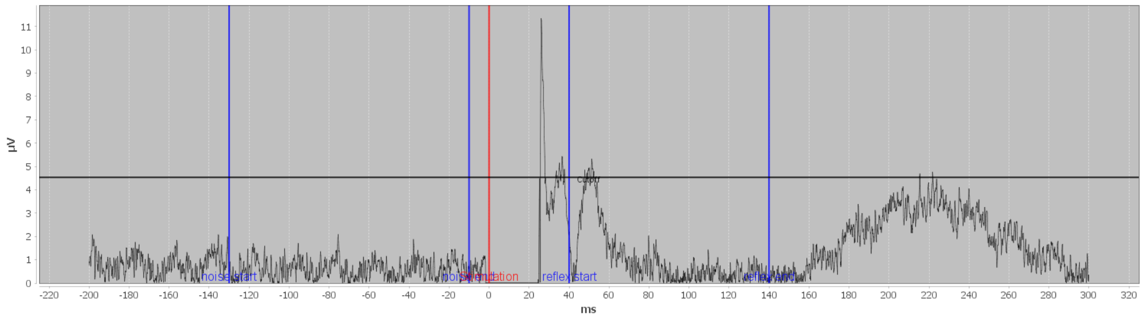

INT

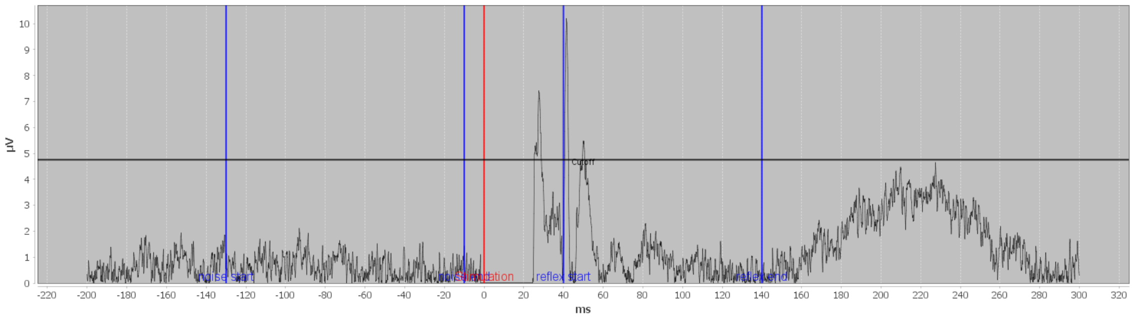

FI

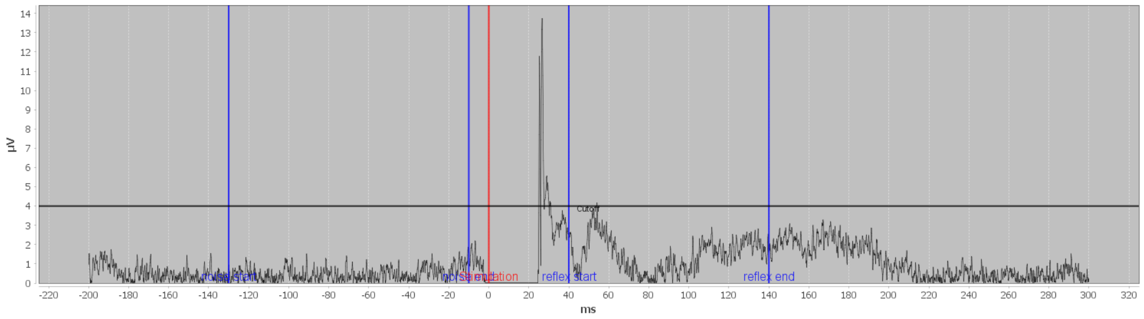

END

P4 – D3

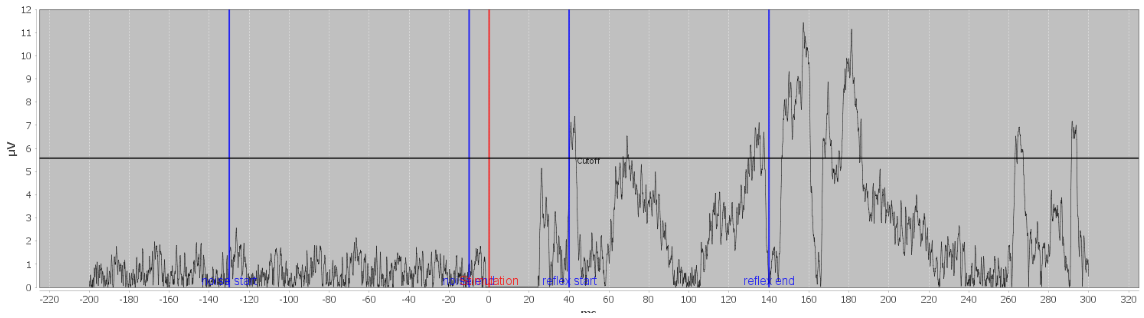

BSL

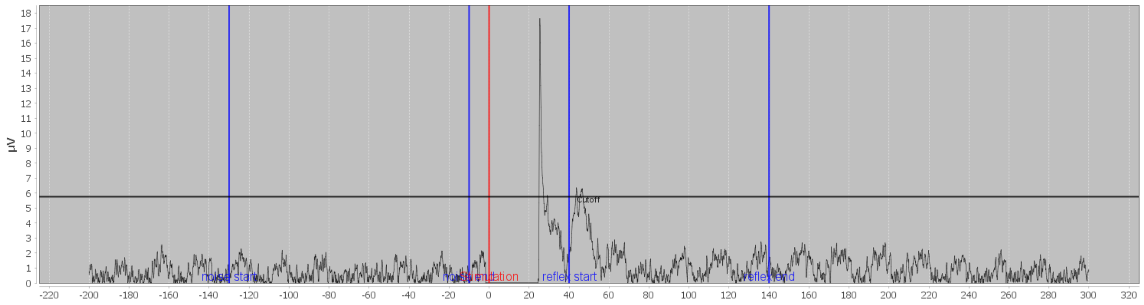

INT

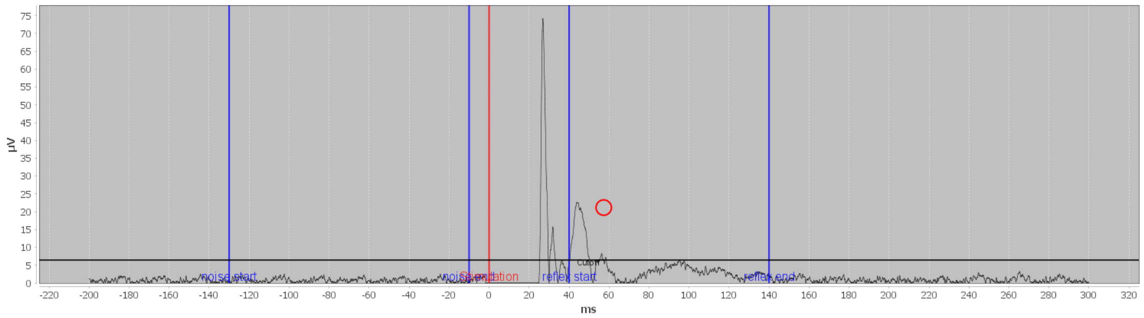

FI

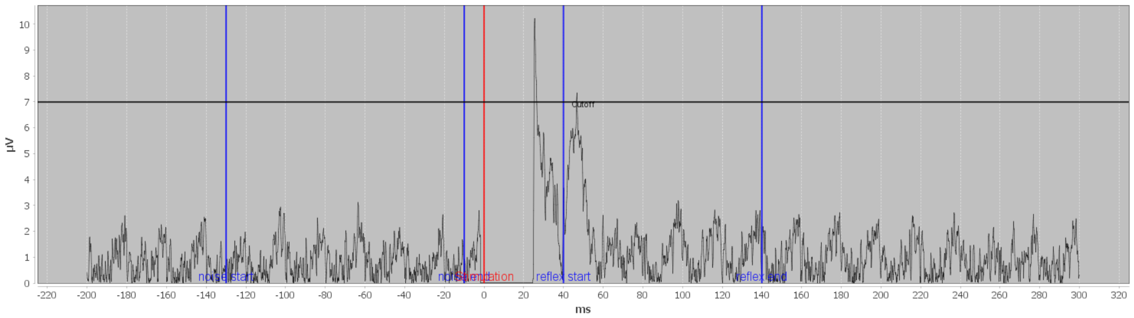

END

P5 – D1

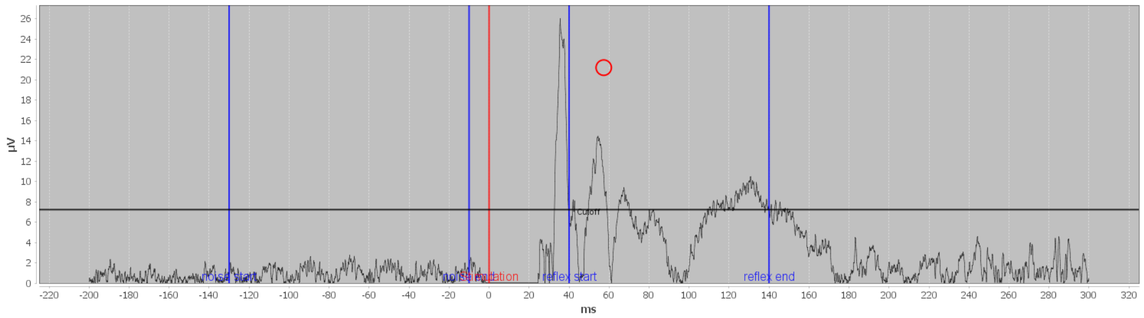

BSL

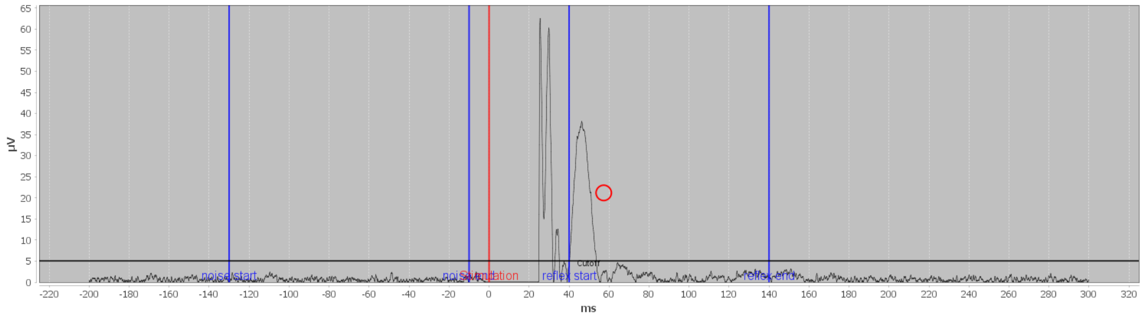

INT

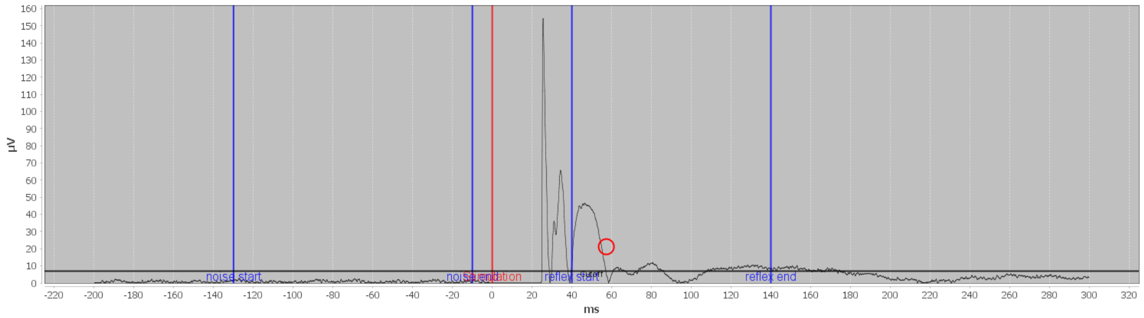

FI

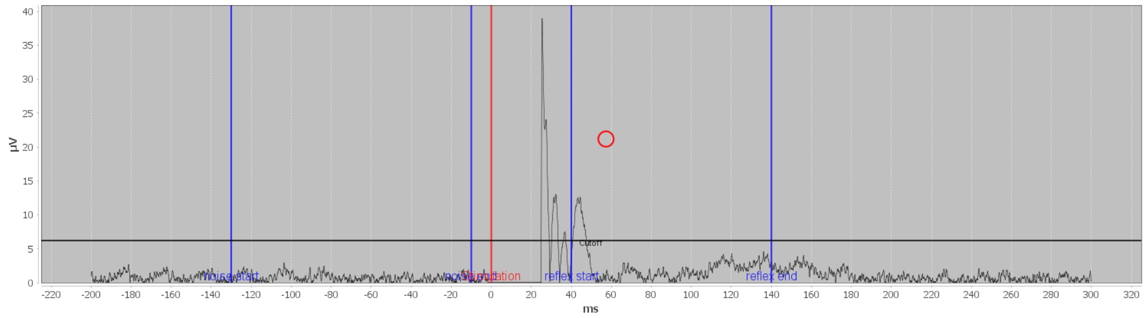

END

P5 – D2

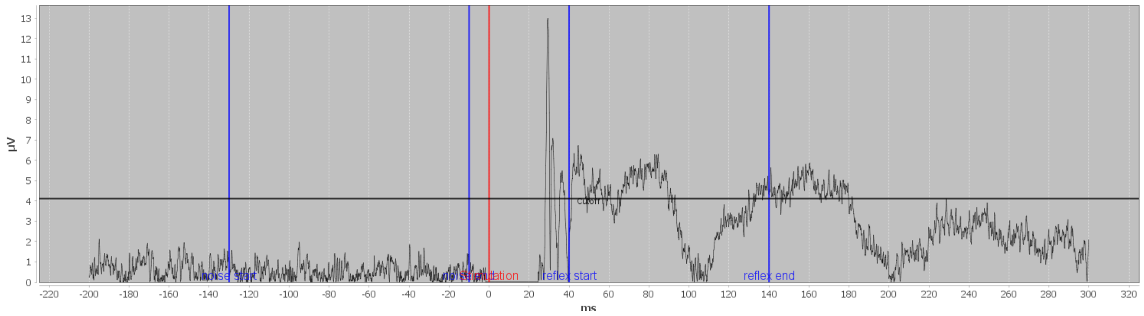

BSL

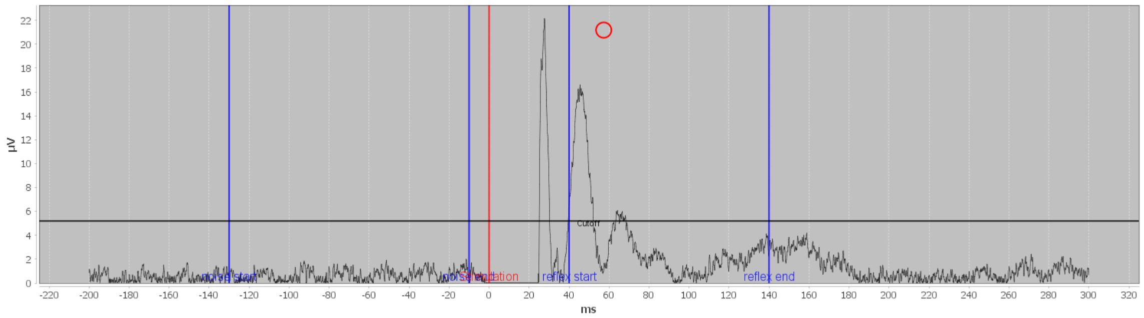

INT

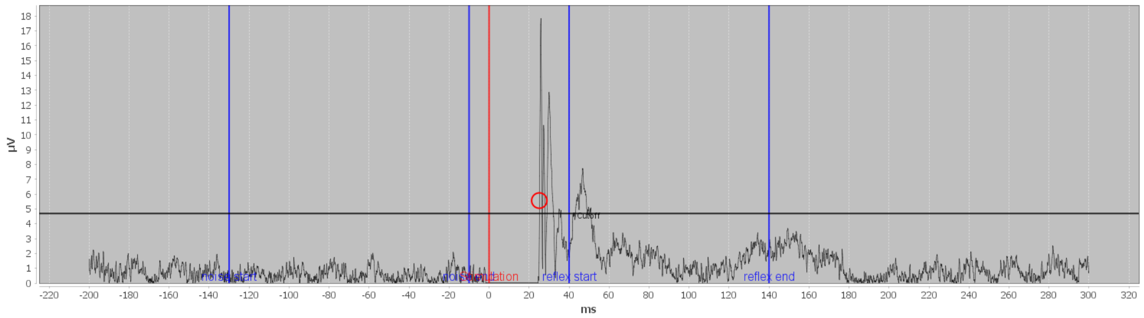

FI

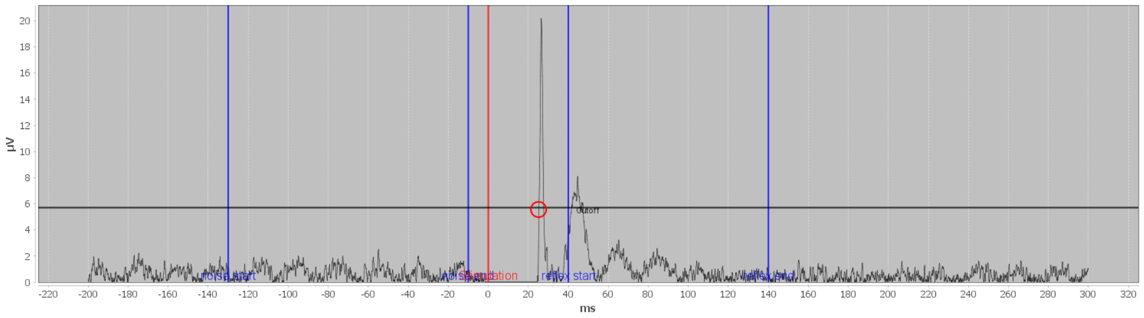

END

P5 – D3

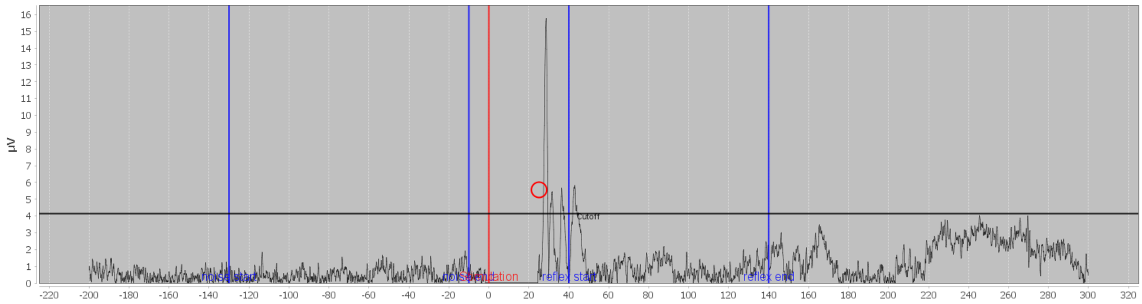

BSL

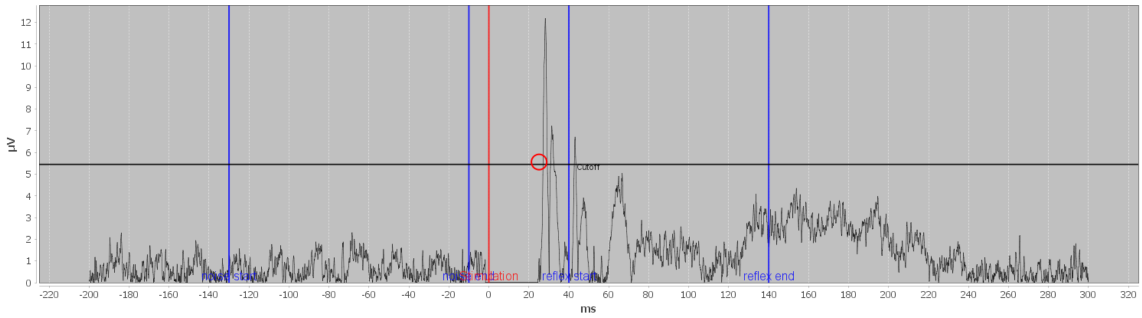

INT

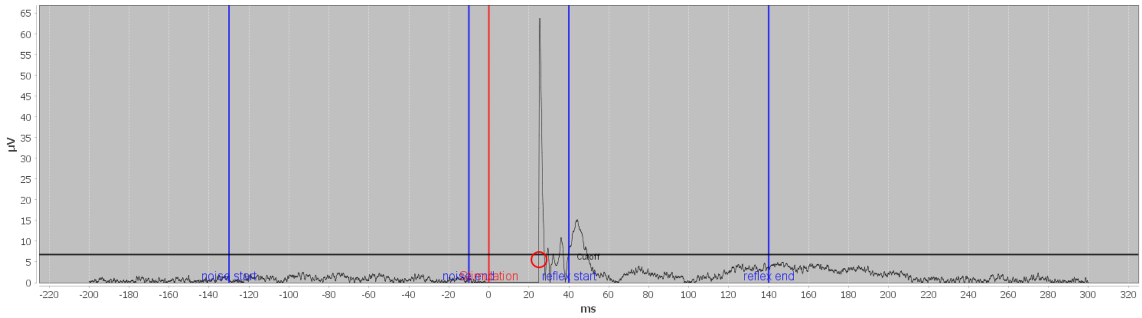

FI

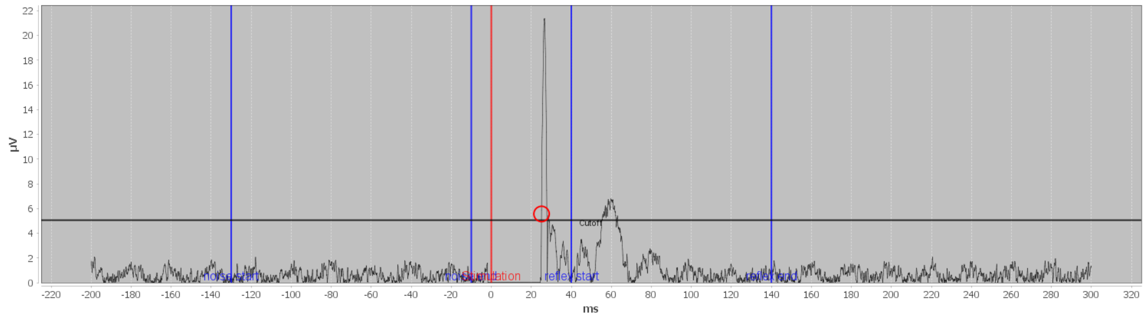

END
