# Supplementary figures and images for: How Is the Nociceptive Withdrawal Reflex Influenced by Increasing Doses of Propofol in Pigs?
Source: Animals (Basel). 2024 Apr 2;14(7):1081. doi: 10.3390/ani14071081 (PMC11010981; doi:10.3390/ani14071081)

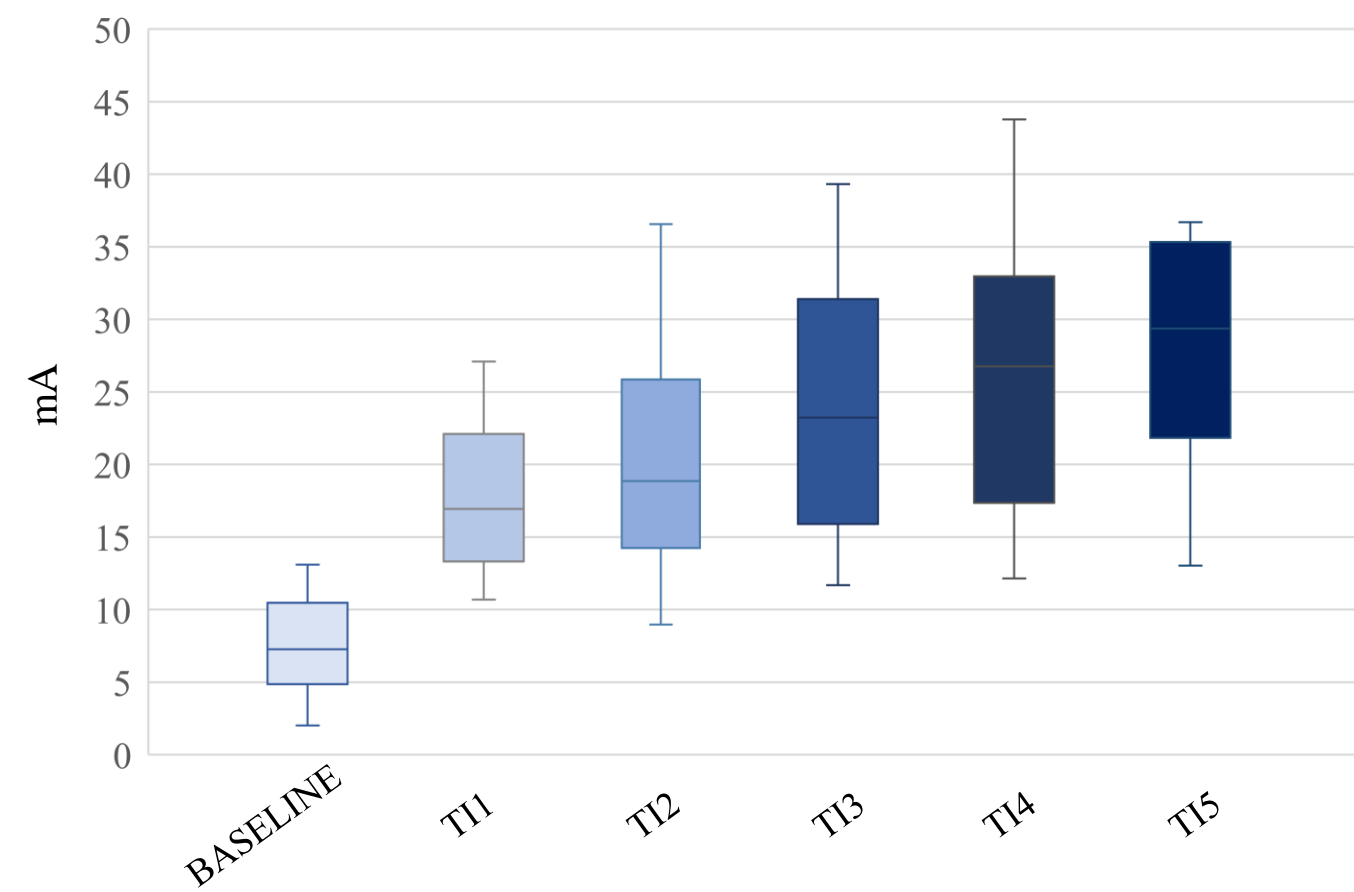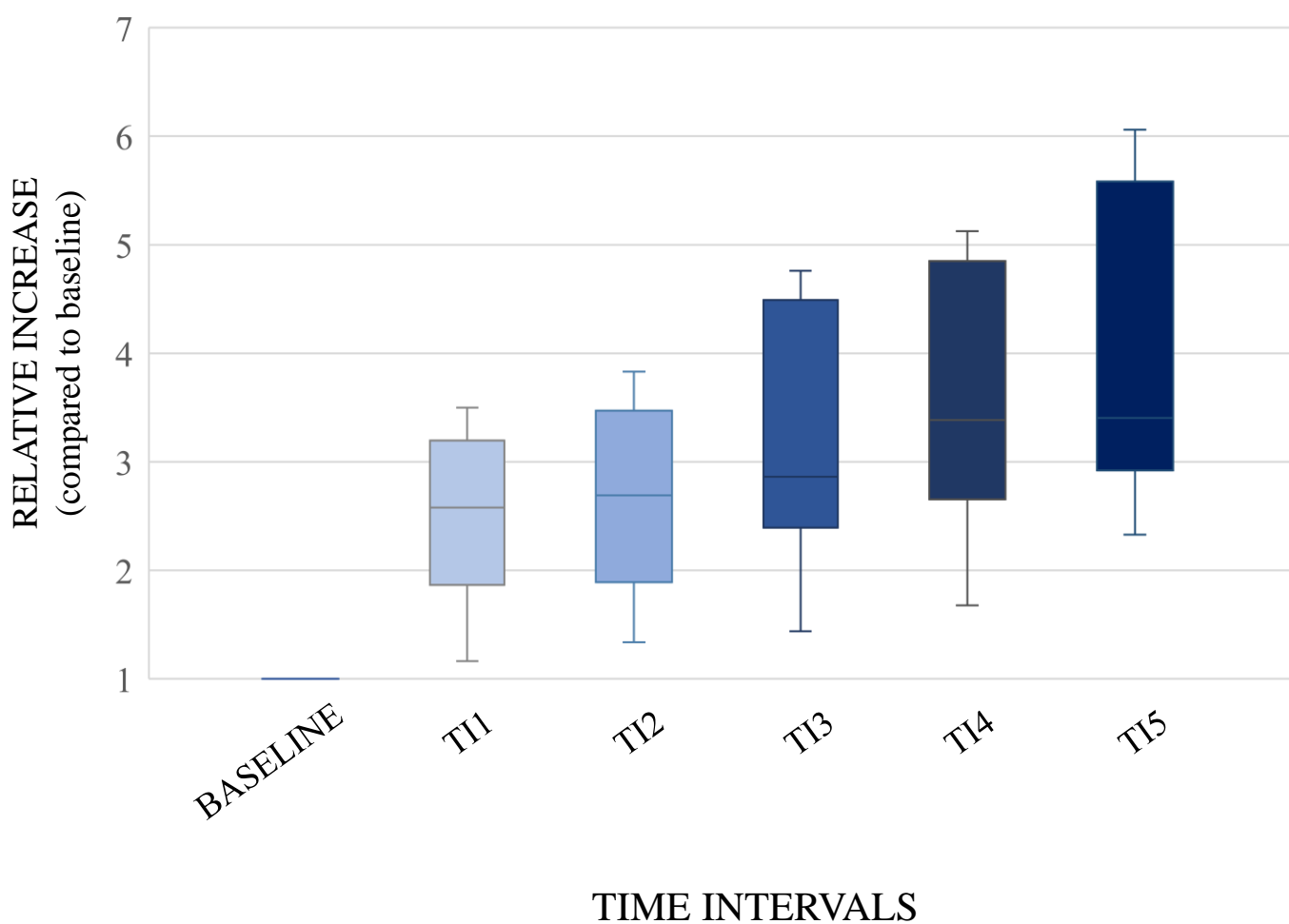

Supplement: Supplementary file 1 [file animals-14-01081-s001.zip › File S1.pdf]

DAY 1

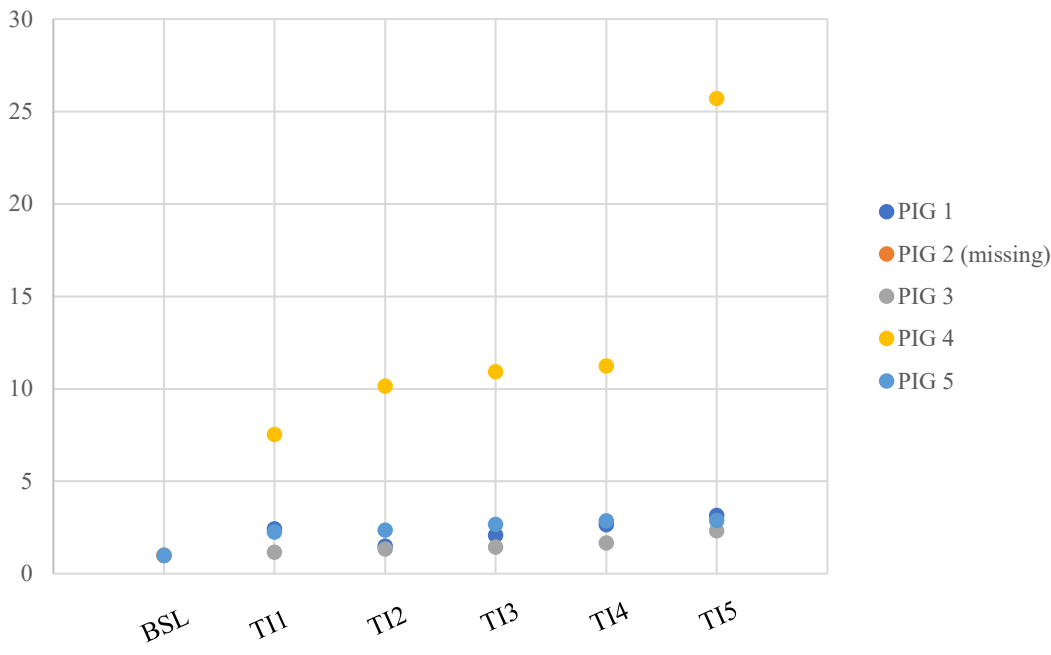

DAY 2

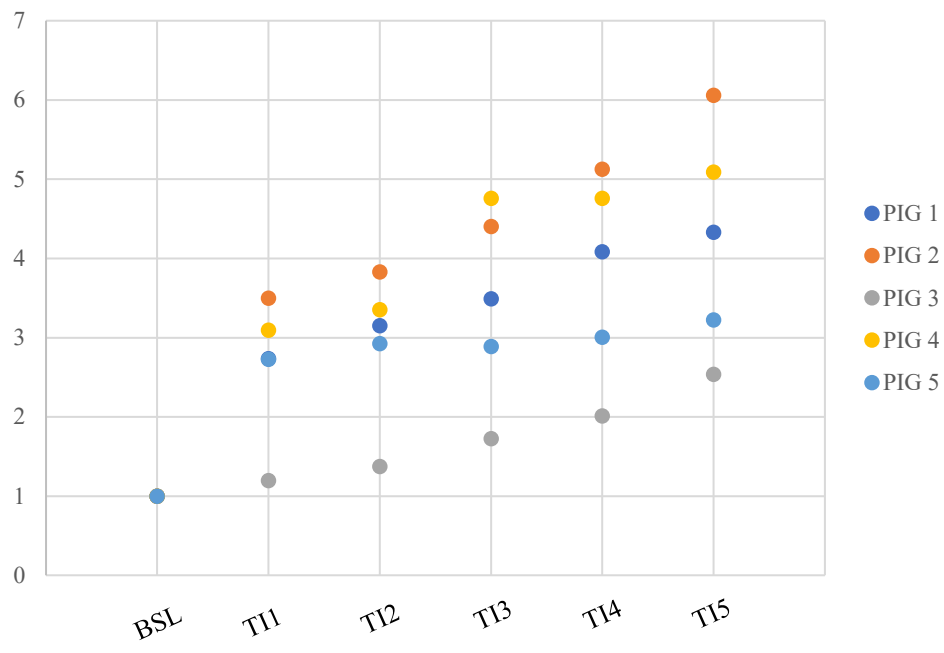

DAY 3

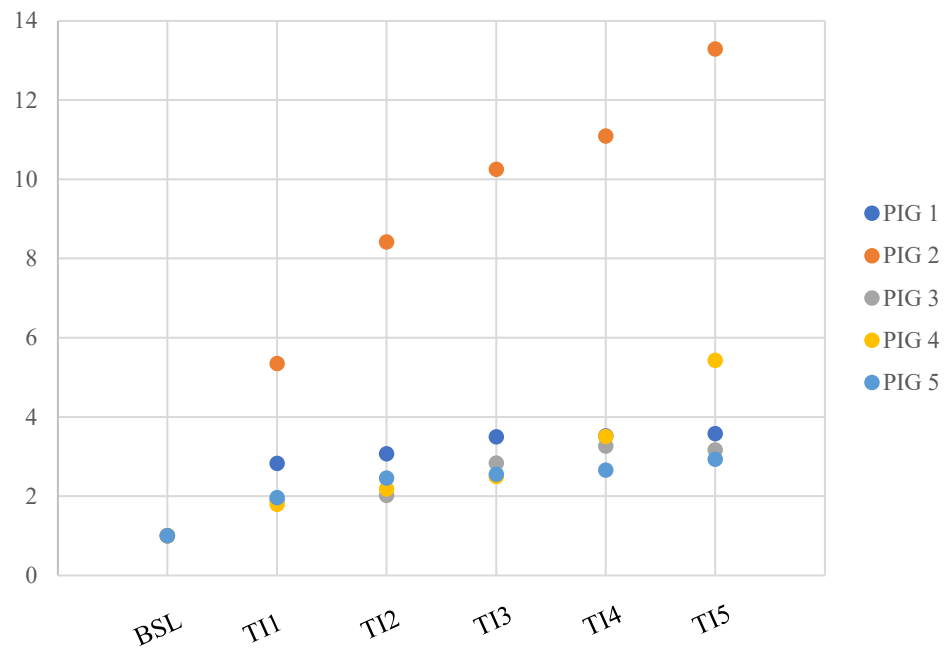

FIG 1

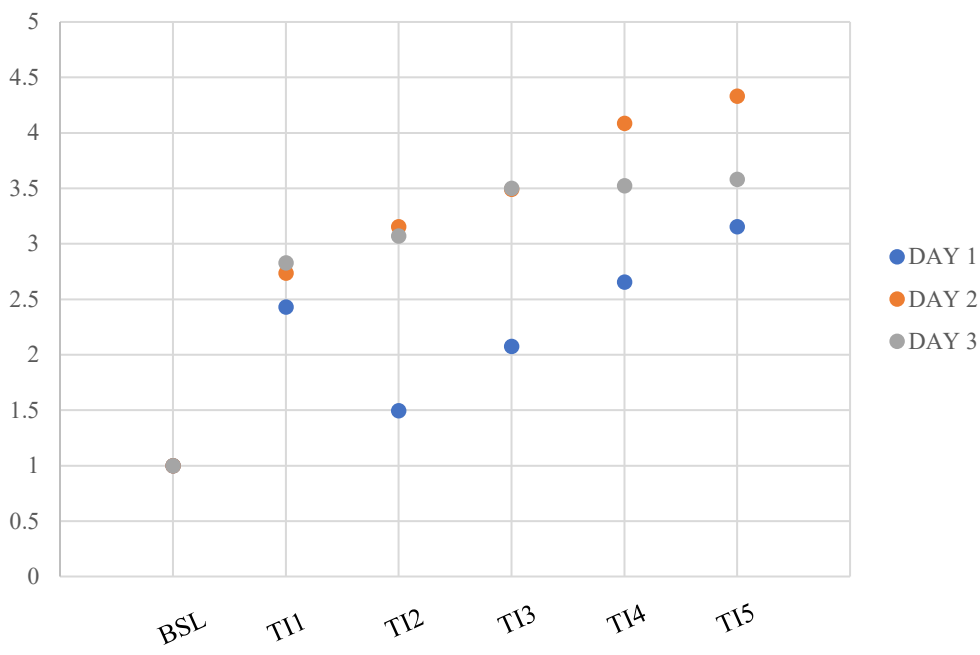

FIG 2

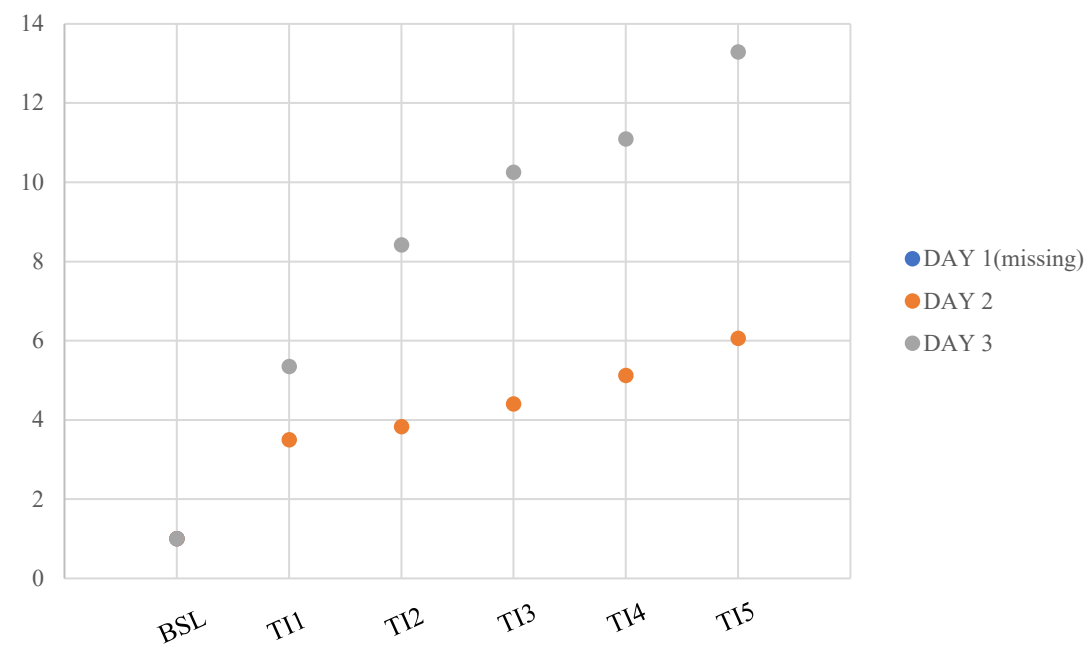

FIG 3

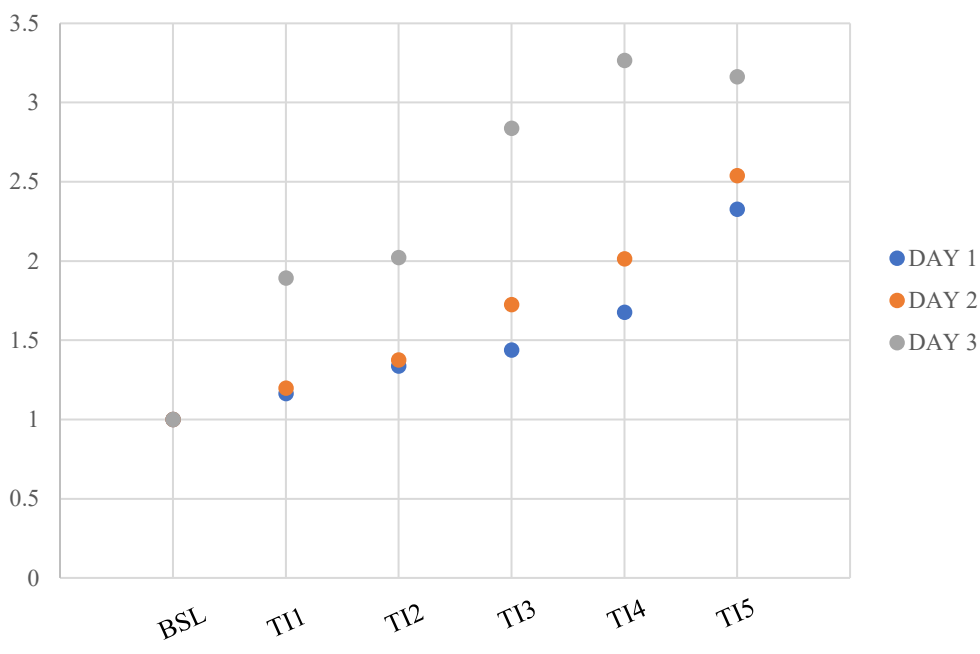

FIG 4

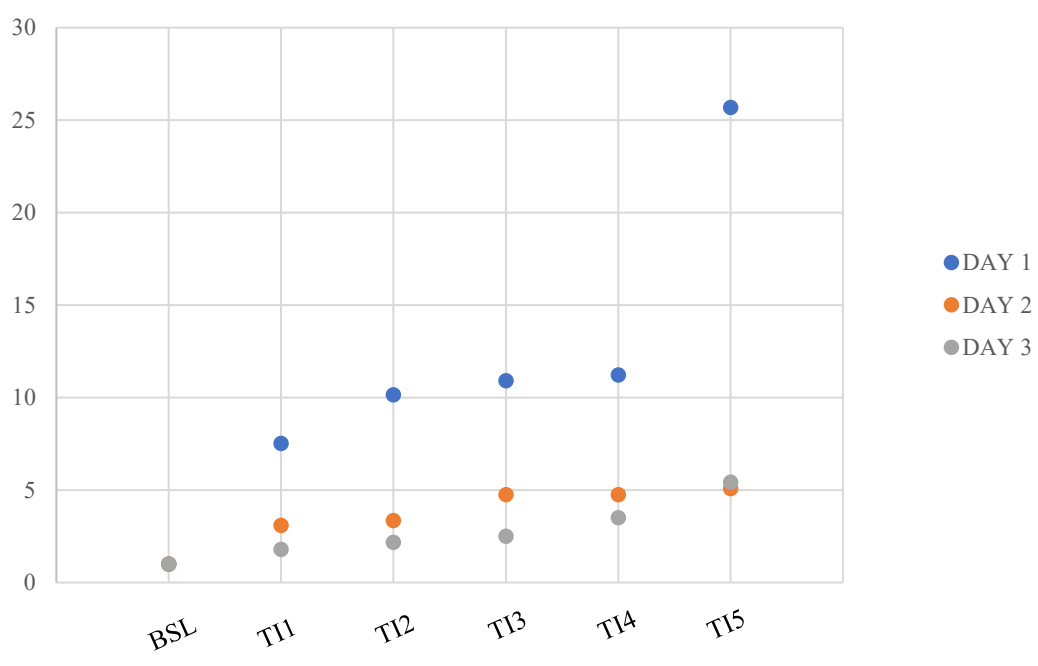

FIG 5

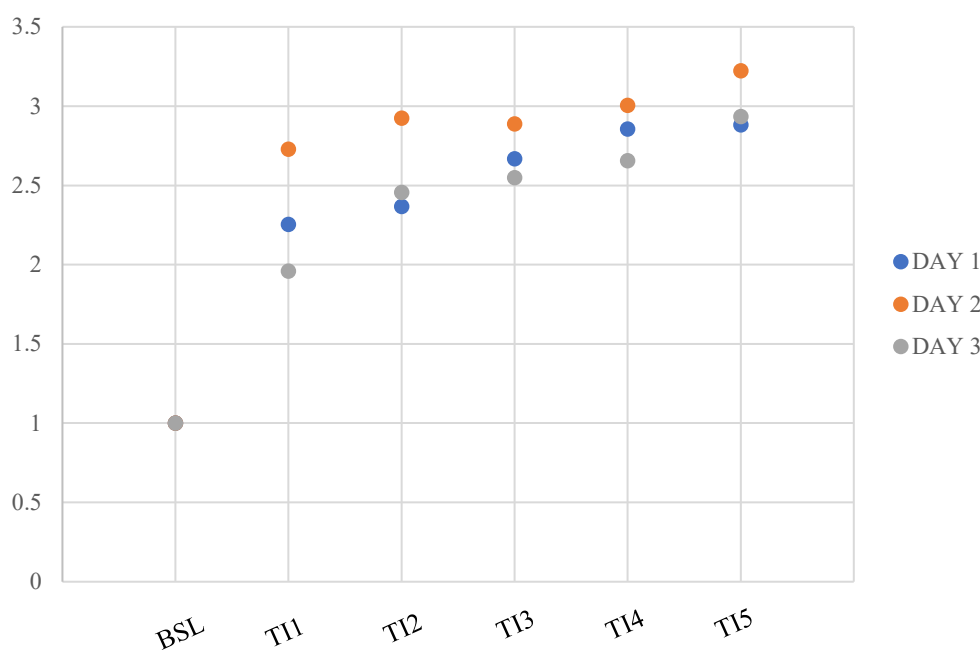

Supplement: Supplementary file 1 [file animals-14-01081-s001.zip › File S3.pdf]
